# Minimal Erythema Dose Determination in Holstein Friesian Cattle

**DOI:** 10.3389/fvets.2021.757452

**Published:** 2021-11-01

**Authors:** Jaka Jakob Hodnik, Marko Jankovec, Jožica Ježek, Žiga Krušič, Stefan Mitterhofer, Jože Starič

**Affiliations:** ^1^Clinic for Reproduction and Large Animals-Section for Ruminants, Veterinary Faculty, University of Ljubljana, Ljubljana, Slovenia; ^2^Laboratory of Photovoltaics and Optoelectronics, Faculty of Electrical Engineering, University of Ljubljana, Ljubljana, Slovenia; ^3^Department of Neurology, General Hospital Celje, Celje, Slovenia

**Keywords:** cow, ultraviolet rays, sunburn, phototesting, hair coat

## Abstract

Cattle on pasture are continuously exposed to solar UV radiation, which has been associated with biological effects such as sunburn, photosensitization, squamous cell carcinoma, and cutaneous vitamin D_3_ production. The minimal erythema dose (MED) required to produce first-degree sunburn (erythema) is poorly researched in cattle. Since cattle are naturally covered with dense hair coats, the MED is influenced by the UV protection offered by the hair. The objective of this study was to determine the MED on intact-hair-covered (MED-H) and shaved white skin (MED-S) of Holstein Friesian cattle. Twenty-one Holstein Friesian cows and heifers were MED tested using a narrowband UV-B LED light (peak irradiance at 292 nm) on eight hair-covered and eight shaved areas over white skin previously unexposed to direct sunlight. Erythema was visually assessed after 24 h. The mean MED-H and MED-S were 5,595 and 329 J/m^2^, respectively. Heifers had a higher MED-H compared to cows, 7,600 and 4,969 J/m^2^, respectively. The mean UV transmittance of white cattle hair was 6.7%. MED-H was correlated with hair length (Spearman's rho = 0.76). A linear regression model showed that each millimeter of hair coat length increased the MED-H by 316 J/m^2^. In conclusion, this study provides a MED testing protocol for cattle and reports standardized values of MED for cattle on intact-hair-covered and shaved areas.

## Introduction

Minimal erythema dose (MED) is defined as the ultraviolet (UV) dose that produces perceptible erythema or erythema with defined boundaries on an individual's skin (1–3). MED is dependent on constitutive skin color, skin thickness, prior UV exposure (thickening of skin and facultative pigmentation), and immune status (4). The dose is usually reported using the International Commission on Illumination (La Commission Internationale de l'Eclairage—CIE) human erythema action spectrum weighting (5), which allows the direct comparison of different UV light sources. MED testing is performed by exposing a skin surface to a range of UV doses and reading the results after 24 h. The lowest dose that produces erythema is the MED for that individual (1–3). Because cattle, like most animals, produce vitamin D_3_ in their skin under the influence of UV irradiation, the MED could be used to determine safe UV exposure dose guidelines, as has been done for humans (6).

The MED of cattle was first reported to be 100 J/m^2^ by Mehlhorn and Steiger (7). However, they did not clearly define the spectral weighting function, which would explain the erythema weighting of the used light source (8). Since then, there have been a small number of studies that exposed cattle to UV radiation and did not detect any erythema at higher doses. Morrow et al. exposed the shaved skin of 10 cows to a dose of 2,430 J/m^2^ UV-B (280–320 nm) (176 J/m^2^ CIE erythema weighted dose) and observed no visual or histological sunburn damage. The study was conducted on Holstein cattle; however, the color of the irradiated skin was not reported (9). In other studies, cows were irradiated with 2,400 J/m^2^ (CIE erythema weighted dose) on unshaven skin and no negative effects were reported (10, 11).

In conclusion, the MED has been poorly studied in cattle and the values remain controversial. The aim of this study was to determine the MED of cattle on intact-hair-covered and shaved white skin with an artificial narrowband UV-B light-emitting diode (LED) light source.

## Methods

### Animals

A total of 21 Holstein cattle from two farms were selected for this experiment. The cattle were either cows (*n* = 16) or older heifers (*n* = 5). The cutoff between cows and heifers was based on having given birth to a calf and being aged 2.5 years or older. The ages of cattle in the study ranged from 22 months to 10.6 years (median = 3.8 years). Cattle were always housed indoors and had no access to direct sunlight. The study was conducted from late April to mid May 2021. The cattle had already shed their winter coats prior to the start of the study. The animals were randomly chosen from the two herds. The only inclusion criterion was that an animal had to have a sufficiently large white area on the dorsal pelvic region to allow the placement of the UV device. The study was approved by the Commission for Animal Welfare at the Veterinary Faculty, University of Ljubljana, on March 25, 2020.

### UV Device

**Figure 1:**
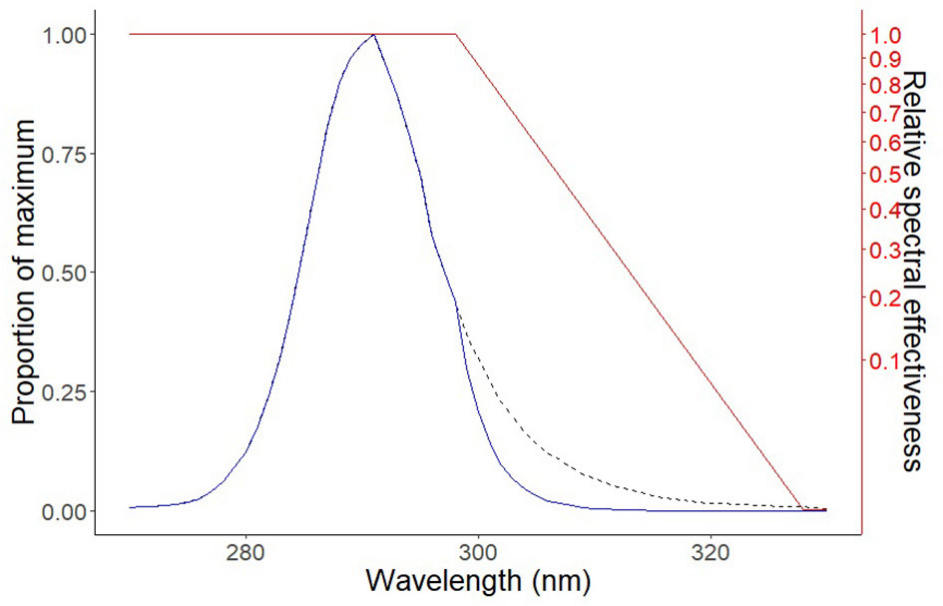
The figure presents the output wavelength curve of the UV light-emitting diode (LED) device and demonstrates the effectiveness of the UV source to produce erythema. The diagram of the spectral output of the UV LED device (black dashed line), the spectral output of the UV LED device weighted by the International Commission on Illumination (CIE) action spectrum (solid blue line), and the CIE action spectrum of human erythema (solid red line) (5).

**Figure 2:**
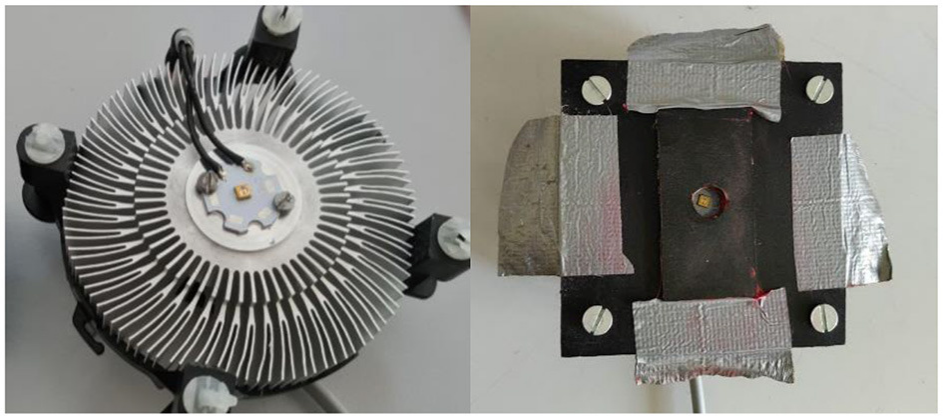
UV LED light mounted on an aluminum cooler (left) and UV LED light with the template (right).

A UV LED Houkem-SMD 3535-290-300 nm (Dongguan Houke Electronic Co., Ltd., GuangDong, China) was used as the UV source. The UV LED emitted 3 mW/cm^2^ of narrowband UV-B light with peak irradiance at 292 nm (Figure 1). The relative spectrum of the emitted light was measured using a spectrometer HR4000 (Ocean Optics Inc., Dunedin, FL, USA), while the absolute total optical power was measured by a 10-W Thermal power meter S310C (Thorlabs, Germany). Using the CIE human erythema action spectrum (5), the erythema-weighted power of the lamp was calculated to be 89.75% (Figure 1). The illumination angle was 120° (range 30–150°). The UV LED was mounted on an aluminum cooler. A black PVC template with a circular opening (diameter 1 cm) was used through which the skin was exposed to UV irradiation (Figure 2). The UV LED was suspended 1 cm above the irradiated surface. The opening in the template limited the illumination angle (60–120°); therefore, only 55% of the total emitted irradiance reached the skin of the cattle. The irradiance of the UV LED light weighted with the CIE action spectrum was therefore 1.48 mW/cm^2^.

### Experiment

**Figure 3:**
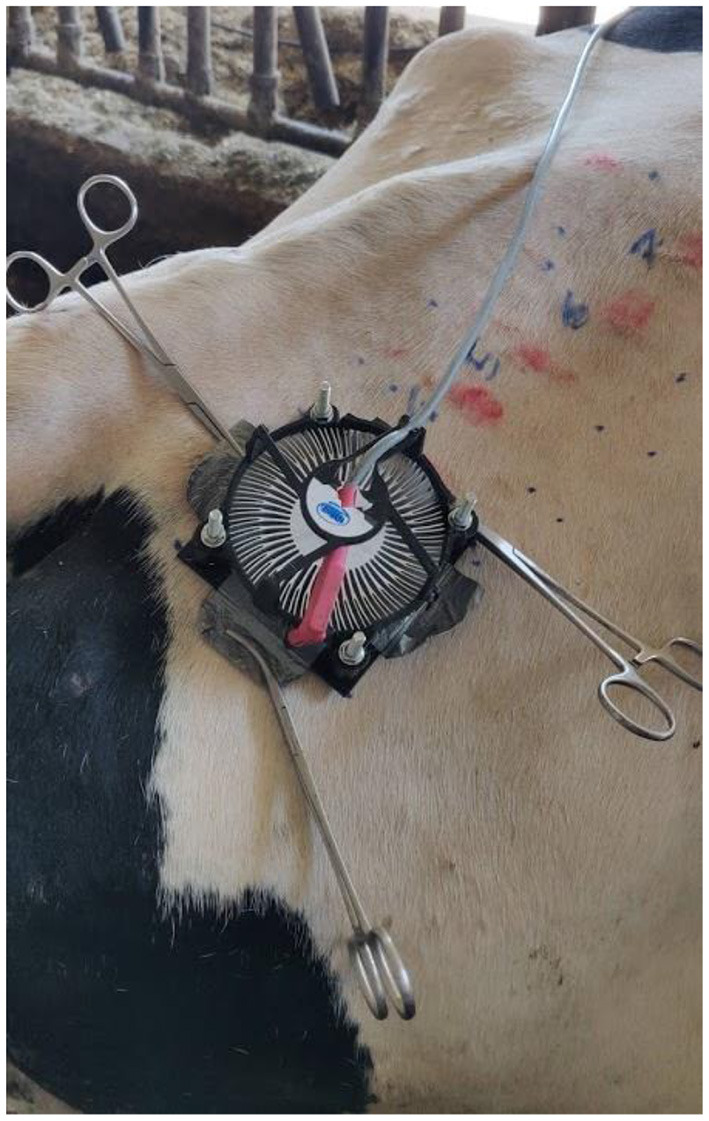
UV light-emitting diode (LED) fixed onto the dorsal pelvis region of cattle with Rochester-Pean forceps.

**Figure 4:**
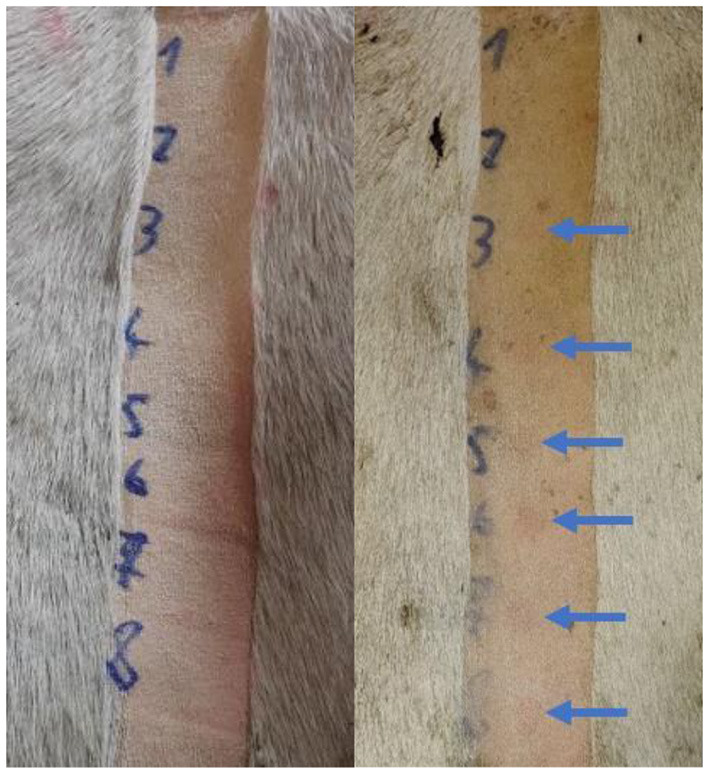
Photograph of the shaved area immediately after irradiation (left) and 24 h after irradiation (right). The perceptible erythema is marked with arrows. The third dose was the lowest dose that still produced perceptible erythema. The minimal erythema dose (MED) on shaved skin for this cow was therefore 300 J/m^2^.

The skin on the dorsal pelvic region was chosen because this region is relatively flat in cattle and is one of the most exposed to UV radiation under natural conditions. The skin was irradiated through hair and on shaved areas. Only white areas were chosen as they are more susceptible to UV and would therefore reflect the true MED of cattle. Eight different doses were used to determine the MED on intact-hair-covered (MED-H) and shaved (MED-S) skin. The hair on the irradiation site was hand brushed into its natural orientation. The UV-LED device was fixed to the hair of the cattle using Rochester-Pean forceps (Figure 3). An electric hair clipper was used to shave the hair, being careful not to injure the underlying skin (Figure 4). The time of irradiation was manipulated to expose the areas to different UV doses. The irradiation protocols are presented in Table 1. The doses were selected based on the information from accessible literature (7, 9–11). Hairs from the irradiation areas were plucked, and the average hair shaft length was calculated. The results of the irradiation were evaluated after 24 h, always by the same two researchers (Figure 4). The lowest dose that produced perceptible erythema was chosen for the MED. The difference between the MED-H and the MED-S was used to calculate the UV transmission through the bovine hair coat.

**Table 1:** Protocol of UV light-emitting diode minimal erythema dose (MED) testing on intact-hair-covered (MED-H) and shaved (MED-S) skin using 1.48 mW/cm^2^ irradiance.

**MED-H**		**MED-S**	
**Dose (J/m^**2**^)**	**Time (s)**	**Dose (J/m^**2**^)**	**Time (s)**
1,500	101	100	7
3,000	203	200	14
4,000	270	300	20
5,000	338	400	27
6,000	405	600	41
7,000	473	800	54
8,000	540	1,000	68
10,000	675	1,500	101

### Statistical Analyses

The statistical analyses and the graph were generated using R statistical software (12, 13). The Shapiro–Wilk test was used to assess the normality of the distribution of the data. The differences between cows and heifers for MED-H and MED-S were analyzed with the Wilcox rank-sum test. The difference of hair length between the two groups was assessed with the Student's *t* test. The correlation between hair length and MED-H was calculated using Spearman's rho correlation coefficient. A linear regression model was fitted to the data to evaluate the association between hair length and MED-H. The residuals of the model were checked for normality. Statistical significance was set at *p* < 0.05.

## Results

**Figure 5:**
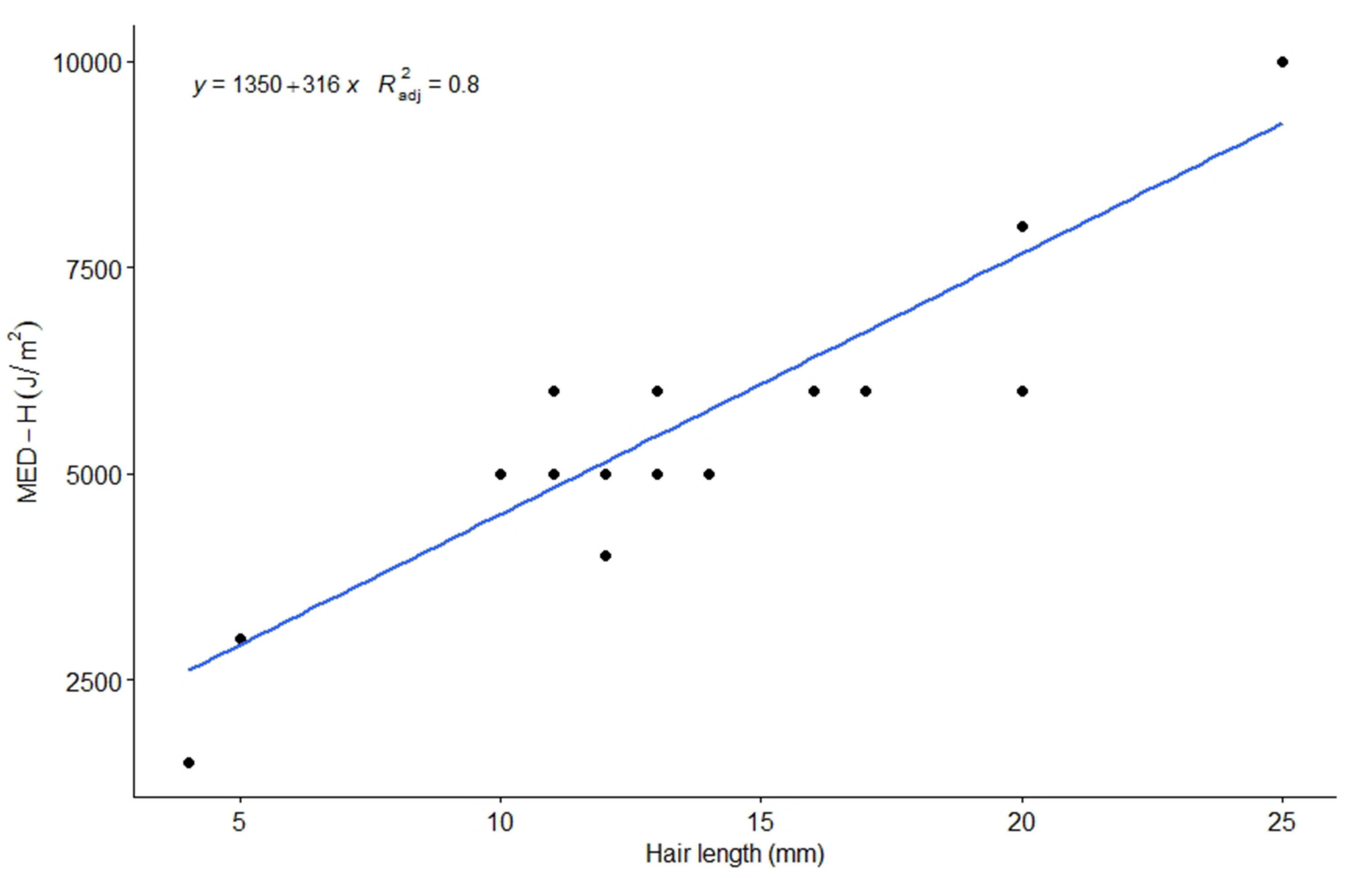
Linear regression model presenting the association between hair length and minimal erythema dose on intact-hair-covered skin (MED-H).

The irradiation produced visible erythema, which resolved spontaneously in 1 week. The animals showed no discomfort during or after the irradiation. The results of UV irradiation are shown in Table 2. The average MED-H was 5595 J/m^2^, while the average MED-S was 329 J/m^2^. Heifers had a higher MED-H compared to cows, averaging 7,600 and 4,969 J/m^2^, respectively (*p* = 0.004). Heifers (mean = 20.2 mm) had longer hair compared to cows (mean = 11.3 mm) (*p* = 0.001). The difference between cows and heifers for MED-S was close to statistical significance (*p* = 0.053), averaging 344 and 280 J/m^2^, respectively. The amount of UV irradiance that passed through the cattle hair was dependent on hair length; the mean value obtained for cattle in this study was 6.7%. The Spearman's rho correlation coefficient for hair length and MED-H was 0.76 (*p* = 0.00006). The linear model explained 80% of the variability (*R*^2^ = 0.8). For each millimeter increase in hair length, the MED-H increased by 316 J/m^2^ (*p* = 3.4^*^10^−8^) (Figure 5).

**Table 2:** Results of UV irradiation.

	**Age (years)**	**MED-H (J/m^**2**^)**	**MED-S (J/m^**2**^)**	**UV transmission through hair (%)**	**Hair length (mm)**
Cow	5.3	5,000	300	6	11
	10.7	6,000	300	6.7	17
	6.5	3,000	300	10	5
	3.8	5,000	400	8	12
	4.3	4,000	200	5	12
	3.9	6,000	400	6.7	11
	5.7	5,000	400	8	10
	5.0	5,000	400	8	14
	2.8	6,000	400	6.7	13
	7.9	5,000	400	8	10
	3.7	6,000	400	6.7	13
	2.8	5,000	300	6	13
	4.9	6,000	300	5	13
	8.5	≤1,500	300	20	4
	2.7	6,000	400	6.7	11
	2.5	5,000	300	6	12
Mean cows	5.0 ± 0.6	4,969 ± 311	344 ± 16	7.6 ± 0.9	11.3 ± 0.8
Heifers	1.8	8,000	200	2.5	20
	2.1	6,000	300	5	16
	1.8	8,000	300	3.8	20
	2.0	6,000	300	5	20
	2.3	10,000	300	3	25
Mean heifers	2.0 ± 0.1	7,600 ± 748	280 ± 20	3.85 ± 0.5	20.2 ± 1.4
Total mean	4.3 ± 0.5	5,595 ± 381	329 ± 14	6.7 ± 0.8	13.4 ± 1.1

*The age of cattle, minimal erythema dose (MED) for intact-hair-covered (MED-H) and shaved (MED-S) skin, the UV transmission through hair, and hair length are presented. Means are reported with ± Standard error of the mean*.

## Discussion

The MED-H of cattle ranged from ≤ 1,500 to 10,000 J/m^2^, with a mean of 5,595 J/m^2^. The observed mean value of MED-H is consistent with previous studies that reported no erythema at a dose of 2,400 J/m^2^ (10, 11). However, one cow with a short hair coat (4 mm) had a MED-H of 1,500 J/m^2^ or lower. The dose of solar radiation reaching the Earth's surface is dependent on latitude, time of day, season, altitude, cloud cover, pollution, and thickness of the ozone layer (14). Daily solar radiation can exceed doses of 5,000 J/m^2^ in summer at mid-latitude locations such as Spain, Germany, Austria, and New Zealand (15–18). In such locations, it is feasible that cattle kept at pasture without access to shelter could be exposed to sufficient UV radiation to reach and exceed their MED-H, leading to sunburn. The areas with sparser hair coat such as the vulva, muzzle, udder, and around the eyes are even more susceptible. Special consideration must be given to cattle with shaved skin areas (e.g., after surgery), as MED-S can be reached considerably faster. The MED-S ranged from 200 to 400 J/m^2^ with a mean of 329 J/m^2^. A dose of 300 J/m^2^ can be reached in half an hour at UV index 6 (150 mW/m^2^).

The mean MED-S is higher than the value reported in the literature (100 J/m^2^); however, the spectral weighting function was not clearly defined in that study (7). Our MED-S is consistent with that of Morrow et al. (9), which found neither visible nor histological sunburn damage at the dose 175 J/m^2^. Our measured cattle MED-S is higher compared to that reported for pigs (165 J/m^2^) and lower compared to that reported for horses (450 J/m^2^) (8). In humans, the MED depends on skin pigmentation and ranges from 200 to 2,000 J/m^2^ for fair- to dark-skinned individuals (19). The white areas of skin and hair in Holstein cattle are the result of the absence of melanocytes in these areas. The pigmentation is called Piebald and is the result of interrupted melanocyte migration to these areas during embryonic development (20, 21). Since melanin is one of the most important UV-protective factors, these areas are more susceptible to UV injury (22). Clear examples of this are photosensitization and the occurrence of eye squamous cell carcinoma in cattle, which usually affect non-pigmented skin (23–25). Histologically, human, porcine, and bovine skin are similar in epidermal thickness (26). Therefore, there must be another explanation besides constitutive skin color, skin thickness, prior UV exposure (thickening of skin and facultative pigmentation), and immune status (4) that would explain the interspecies variability in MED. Endogenous UV-protective factors in the skin, such as antioxidants (carotenoids, vitamins E and C, polyphenols) and micronutrients (selenium), have an effect on MED and could explain the difference (27). The latter could also explain the almost significant difference in the MED-S between heifers and cows as the feed differs between these two categories.

The density of hair coverage in heifers is greater than that in cows, due to the number of hair follicles being fixed at birth and the resultant spread of this number of follicles over a larger area as the animal grows (28, 29). The denser the cattle hair, the lower the UV transmission through that hair (8). In this study, MED-H was correlated with hair coat length. Both of these factors could explain the observed higher mean MED-H values (lower UV transmission through hair) for heifers (7,600 J/m^2^) compared to cows (4,969 J/m^2^) (*p* = 0.0037). Hair density also varies between different body regions, with the densest hair found on the shoulders (28, 30). The density and length of cattle hair varies seasonally, with both parameters peaking in winter (28, 30). In our study, the observed UV transmittance of cattle hair ranged from 2.5 to 20%, with a mean value of 6.7% these findings similar to the values observed in an earlier study (31). MED-H was determined only on the dorsal pelvis area in our study; therefore, the true MED of unshaved cattle could be lower in other regions of the cow with a thinner hair coat. However, the dorsal pelvis was chosen in this study because it is relatively flat and the back of cattle on pasture is naturally the area most exposed to UV radiation.

Cattle, like humans, produce Vitamin D_3_ in their skin under the influence of UV irradiation. In humans, there is a guideline for safe UV exposure to meet daily vitamin D needs, called Holick's rule, which states that humans have to expose one-fourth of their skin surface area to one-fourth of their MED each day to meet their needs (6). Further research is required to establish whether this is also true for cattle. To date, there is evidence that the application of approximately one-fourth (1,200 J/m^2^) of our determined MED-H to an unknown portion of the skin surface area of cows (irradiated from behind) was enough to maintain vitamin D levels above sufficiency (25-hydroxyvitamin D >30 ng/ml) (11, 32). Elsewhere, ~1/3 (1,800 J/m^2^) of our determined MED-H applied to cattle *via* natural conditions on pasture was enough to sustain blood vitamin D concentrations (33). UV wavelengths between 295 and 300 nm have been found to be optimal for vitamin D_3_ production in humans (34), although a study on human skin samples using different wavelengths of UV LED light reported similar production even at lower wavelengths (close to the peak wavelength of the light used in this study, 292 nm) (35). Therefore, using our results as a guide, similar UV LED lights could potentially be used to stimulate sufficient production of vitamin D in cattle without causing skin injury. The long-term safety implications would, however, require further investigation before such an approach could be recommended.

The main limitation of this study was the use of a narrowband UV-B irradiation source, as wavelength affects the transmittance of UV light, with shorter wavelengths having lower transmittance through white hair (36). The use of a device that generated higher wavelengths may have demonstrated erythema in the studied cattle under lower UV dosage (due to better transmission of higher wavelengths); however, this effect may have been counteracted by the lower erythema efficiency of such higher wavelengths (5). Additional limitations are the lack of measurement of hair density at the irradiation sights and the determination of the MED on only one body region, as hair density influences the amount of UV radiation transmitted to the skin (8, 36). The results would be more meaningful if presented alongside a measure of hair density. As hair density and length vary on different parts of the body (28, 30), the MED-H of cattle could be similarly variable depending on the body region under consideration. Use of smaller dose intervals would allow for a more accurate estimate of the MED. The use of the cattle-specific erythema action spectrum, which may differ from that for humans, and the use of a spectrophotometer to determine erythema would provide more detailed and objective erythema detection and MED values.

Further studies are needed to determine the MED on other parts of the body with thinner hair coat and to quantify the effect of hair coat density on MED. The effect of endogenous UV-protective factors other than pigment and skin thickness on MED in cattle could also be studied. The same experiments can be conducted on cattle of other breeds to determine how different breed skin and hair characteristics affect the MED. The cattle in this study were housed without access to direct sunlight their entire lives. Therefore, a study of how Piebald pigmented skin adapts to repeated UV exposure (e.g., hyperkeratosis, thickening of the epidermis) would also be interesting. The cattle erythema action spectrum has not yet been determined and may also be the focus of future studies.

## Conclusions

The presence of hair and hair length influenced MED values in cattle. The MED value increased by 316 J/m^2^ with each additional millimeter of hair length. Heifers were observed to have longer hair coats and higher MED values for intact-hair-covered skin than cows. MED values for shaved skin did not differ between cows and heifers.

## Data Availability Statement

The raw data supporting the conclusions of this article will be made available by the authors, without undue reservation.

## Ethics Statement

The animal study was reviewed and approved by Commission for Animal Welfare at the Veterinary Faculty, University of Ljubljana. Written informed consent was obtained from the owners for the participation of their animals in this study.

## Author Contributions

All authors contributed to the design, writing, and editing of the manuscript.

## Funding

This study was partially financed by the young researcher program and Program group P4-0092 (Animal Health, Environment and Food Safety) of the Slovenian Research Agency.

## Conflict of Interest

The authors declare that the research was conducted in the absence of any commercial or financial relationships that could be construed as a potential conflict of interest.

## Publisher's Note

All claims expressed in this article are solely those of the authors and do not necessarily represent those of their affiliated organizations, or those of the publisher, the editors and the reviewers. Any product that may be evaluated in this article, or claim that may be made by its manufacturer, is not guaranteed or endorsed by the publisher.
